# Efficacy and Safety of 6-Weekly versus 12-Weekly Intravenous Methylprednisolone in Moderate-to-Severe Active Thyroid-Associated Ophthalmopathy

**DOI:** 10.3390/jcm12093244

**Published:** 2023-05-01

**Authors:** Kenneth K. H. Lai, Fatema Mohamed Ali Abdulla Aljufairi, Chi Lai Li, Amanda K. Y. Ngai, Carly S. K. Yeung, Ryan H. Y. Fong, Wilson W. K. Yip, Alvin L. Young, Chi Pui Pang, Kelvin K. L. Chong

**Affiliations:** 1 Department of Ophthalmology, Tung Wah Eastern Hospital, Hong Kong, China; 2 Department of Ophthalmology and Visual Sciences, Faculty of Medicine, The Chinese University of Hong Kong, Hong Kong, China; 3 Department of Ophthalmology, Salmaniya Medical Complex, Government Hospitals, Manama 323, Bahrain; 4 Department of Ophthalmology and Visual Sciences, Prince of Wales Hospital, Hong Kong, China; 5 Hong Kong Eye Hospital, 147K Argyle Street, Kowloon, Hong Kong, China

**Keywords:** dysthyroid optic neuropathy, intravenous methylprednisolone, thyroid-associated orbitopathy, steroid, thyroid eye disease, Graves’ ophthalmology, autoimmune thyroid disease, biologics, glucocorticoid, euthyroid Graves orbitopathy

## Abstract

Purpose: To compare the efficacy and safety of 6-weekly and 12-weekly intravenous methylprednisolone (IVMP) regimens in moderate-to-severe, active thyroid-associated orbitopathy (TAO) patients. Basic Procedures: Retrospective comparative study of patients who received IVMP between January 2011 and July 2021 at the Thyroid Eye Clinic, the Chinese University of Hong Kong. Outcome measures included the 7-item clinical activity score (CAS), exophthalmos, extraocular muscle motility (EOMy), marginal reflex distance (MRD), best corrected visual acuity (BCVA), intraocular pressure (IOP), the requirement of additional treatment, and complications. Main findings: A total of 65 (63% (41/65) females) moderate-to-severe, active TAO patients aged 50 ± 13 (25–74) years received 6-weekly (n = 22) or 12-weekly (n = 43) IVMP. Sex, age, smoking status, and Graves’ disease status were comparable in the two groups (all *p* > 0.05). CAS at week 6 (*p* = 0.0279), 12 (*p* = 0.00228), and 52 (*p* = 0.0228) were lower at each time for the 12-weekly group. Exophthalmos improved more at week 6 (*p* = 0.0453) and 12 (*p* = 0.0347) in the 12-weekly group. The improvement of diplopia, MRD1, MRD2, and EOMy were comparable between the two groups. More patients in the 6-weekly group (*p* = 0.00169) required additional treatments including IVMP+/−ORT. Patients in the 6-weekly group who did not require additional treatment had a lower presenting CAS (*p* = 0.0193) than those who required additional treatment. The total numbers of adverse events were comparable between the two groups.

## 1. Introduction

Thyroid-associated orbitopathy (TAO), also known as Graves’ ophthalmology (GO) and thyroid eye disease (TED), is an extrathyroidal manifestation of autoimmune thyroid disease (AITD), which may also occur in euthyroid patients as euthyroid Graves orbitopathy (EGO) [1,2]. TAO is diagnosed clinically with characteristic features such as upper eyelid retraction, lid lag, exophthalmos, and conjunctival injection [1]. TAO is the most common orbital disorder in adults with an incidence rate of 5 in 100,000 person-years, and it is more likely to occur in the female sex [3,4]. Risk factors of TAO included female sex, advanced age, smoking, and radioactive iodine therapy [5]. It typically runs a biphasic course, which begins with progressive inflammation that peaks before a stable fibrotic phase [6]. Early detection and treatment are important, since sight-threatening conditions such as exposure keratopathy and dysthyroid optic neuropathy (DON) may develop [7].

Orbital inflammatory infiltration occurs during the active stage of TAO, and the degree of lymphocytic and macrophage infiltration was found to be correlated with the disease activity [8,9]. In mild TAO patients, conservative management including lubricating eye drops may be sufficient to relieve eye symptoms. Selenium supplement was found to improve the soft tissue signs, TAO related quality of life and slow down the progression of the disease [10]. Intravenous glucocorticoid is the first-line treatment in moderate-to-severe, active TAO patients, due to the anti-inflammatory and immunosuppressive actions [11]. The oral route of glucocorticoid is also effective, despite being less efficacious and less well tolerated than the intravenous route [12]. The European Group on Graves’ Orbitopathy (EUGOGO) recently recommended two regimens of intravenous methylprednisolone (IVMP) with cumulative doses of 4.5 g and 7.5 g as the first line of treatment for moderate-to-severe, active TAO [13]. Different regimens of IVMP have been studied. Of note, both the efficacy and safety were considered to be dose-dependent [14,15,16].

High-dose IVMP is administrated under adequate monitoring and medical support. Severe adverse events such as cardiovascular and liver failures have been reported in TAO patients receiving high-dose IVMP [17,18]. The optimal IVMP regimen is still debatable. It is important to balance the indications, dose-related efficacies and complications during IVMP prescription. This study aims to compare and report on the efficacy and safety of the two IVMP regimens: a lower dose 6-weekly and a higher dose 12-weekly regimen in moderate-to-severe, active TAO patients.

## 2. Methods

This is a retrospective comparative study of TAO patients managed at the Thyroid Eye Clinic, the Chinese University of Hong Kong between 1st January 2011 and 1st July 2021. This study followed the Declaration of Helsinki and ethical approval was obtained from the Institutional Review Board (Kowloon Central/Kowloon East-10-0218/ER-3 and Joint Chinese University of Hong Kong-New Territories East Cluster 2010_594). We included patients with the following inclusion criteria:

(1) Clinical diagnosis of TAO^13^
(2) TAO patients who were medically indicated for IVMP regimen
(3) No prior or concurrent use of other immunosuppressive therapy or orbital radiotherapy
(4) No prior or concurrent history of orbital disease, trauma, or surgery
(5) Clinical activity score (CAS) ≥3 on presentation [19]
(6) Moderate to severe TAO^13^ with at least one of the following: palpebral retraction >2 mm, moderate or severe soft tissue involvement, exophthalmos >3 mm, inconstant or constant diplopia, dysthyroid optic neuropathy or exposure keratopathy
(7) Without medical comorbidities that are contraindicated for systemic steroid including cardiac arrhythmia, unexplained epigastric pain, active hepatitis C, human immunodeficiency virus or tuberculosis infection, uncontrolled hypertension, and deranged liver function

The two IVMP regimens:

The 6-weekly IVMP regimen treatment protocol: 3-weekly 500 mg IVMP succinate diluted in 250 mL normal saline for slow infusion over 2 h, followed by 3-weekly 250 mg IVMP succinate diluted in 250 mL normal saline for slow infusion over 1 h. The 12-weekly IVMP regimen treatment protocol: 6-weekly 500 mg IVMP succinate dilated in 500 mL normal saline for slow infusion over 2 h, followed by 6-weekly infusions of 250 mg IVMP succinate dilated in 250 mL normal saline for slow infusion over 1 h.^13^ Sequential sampling of TAO patients who received the 2 IVMP regimens was performed. It was our team’s usual practice to prescribe the 6-weekly IVMP regimen between 2011 and 2013, and the 12-weekly IVMP regimen since 2014. The decision for additional treatment after the first course of IVMP was based on inadequate clinical response (static or worsening of CAS) by the corresponding author.

## 3. Outcomes Measures

The 7-item CAS^13^ was used to assess the clinical activity in TAO patients including on a scalee of 0 to 7, including (1) spontaneous orbital pain, (2) gaze-evoked orbital pain, (3) eyelid swelling, (4) eyelid erythema, (5) conjunctival redness, (6) chemosis, and (7) inflammation of the caruncle or plica. The best corrected visual acuity (BCVA) was documented using the Snellen chart at 6 m. Intraocular pressure (IOP) was measured by applanation tonometry. The Gorman diplopia scale [20] was used to score the diplopia as follows: (1) no diplopia, (2) gaze-evoked diplopia, (3) intermittent, primary-gaze diplopia, and (4) constant primary-gaze (intractable) diplopia or no diplopia due to large angle strabismus. Extraocular motility (EOMy) restriction was scored according to the position of the limbus at 9 cardinal gaze photos. We scored 0 for the full excursion and −5 for failure to reach the midline (−4 to −1 for an excursion in 25% increments) [21]. Exophthalmos was measured by the Hertel exophthalmometer. We monitored the complications related to IVMP administration with the endocrinologists. We checked the body weight and blood pressure before and cardiac monitoring during IVMP administration. Blood samples for serum glucose, lipid profile, and liver functions were performed before, during and after IVMP. Patients receiving other immunosuppressants were jointly monitored by ophthalmologists and endocrinologists, and the immunosuppressants dosages were titrated according to patient’s tolerability and side effects.

Medical records were independently reviewed by 2 oculoplastic surgeons. Neither reviewer was aware of the information retrieved by the other. A consultant oculoplastic surgeon (KC) was involved in the resolution when the results assessed by the two reviewers were different. The outcome measures at the week 6, 12, 25 and 52 were compared to the baseline. Statistical analyses were performed using SPSS statistical software package (Window version 24.0; IBM Corp. in Armonk, NY, USA). Comparison of the treatment outcomes was analyzed using paired T-tests and the Chi-square/Fisher’s exact test, respectively. Statistical significance was defined as *p* < 0.05 Results were expressed as the mean ± standard deviation (range).

## 4. Results

A total of 65 (63% (41/65) females) patients aged 50 ± 13 (25–74) years were reviewed. Twenty-two were treated by the 6-weekly regimen and 43 by the 12-weekly regimen) for moderate-to-severe, active TAO. Fourteen were current smokers and 7 were ex-smokers. There is no significant difference between the age, sex, smoking status, time from diagnosis to treatment, and thyroid disease status between the 2 groups. The baseline clinical presentation including CAS, BCVA, IOP, MRD1, MRD2, exophthalmos, EOMy, and diplopia are comparable between the 2 groups. The baseline information of patients was summarized in Table 1.

We compared the treatment outcomes of both 6-weekly and 12-weekly regimens. There was better improvement of CAS at the 6th, 12th and 52th weeks when compared with the baseline in the 12-weekly group (*p* < 0.05). The improvement of exophthalmos was superior at the 6th and 12th weeks among the 12-weekly group (*p* < 0.05). The percentage of TAO patients who required additional treatment (IVMP, ORT or immunosuppressant) following the course of IVMP was higher among the 6-weekly group (*p* < 0.05). The comparison of treatment outcomes between the 2 groups were summarized in Table 2.

We compared the 14 TAO patients who did not receive additional treatments to the 8 patients who did, the former group was associated with a lower presenting CAS (3 vs. 4, *p* < 0.05) (Table 3). There were no significant differences in adverse events between the 2 groups, and no significant adverse event was reported. The complications of both groups were summarized in Table 4.

## 5. Discussion

In this study, we report on the efficacy and safety of the 6-weekly IVMP regimen, which consists of half of the cumulative dose and treatment interval of the 12-weekly IVMP regimen, as recommended by the EUGOGO clinical practice guidelines [13]. The 12-weekly group showed superior improvement in both the CAS and exophthalmos at the 6th and 12th weeks of treatment. Despite a higher proportion of the 6-weekly IVMP group requiring additional treatment due to inadequate clinical response, importantly, we showed that those who did not require additional treatment were associated with a lower presenting CAS. It is not surprising that more patients with higher presenting CAS required additional treatment, and therefore, the role of activity-based individualized IVMP regimens for TAO patients should be explored.

TAO is typically diagnosed through a combination of clinical examination and radiological testing. However, varying signs of TAO can be overlooked, and up to 25% of TAO patients had a minimum of a year delay in the diagnosis [22]. The management is usually guided by both disease activity and severity, and the CAS is commonly adopted to assess the TAO activity. It involved evaluating the degree of eyelid inflammation, swelling, redness, and chemosis, as well as the severity of spontaneous and gaze-evoked pain on eye movement [23]. The short tau inversion recovery (STIR) sequence on the coronal plane of magnetic resonance images (MRI) was also reported as a sensitive radiologically marker for disease activity, by comparing the signal intensity of individual extraocular muscle with the ipsilateral temporalis muscle to give the signal intensity ratio (SIR) [24]. Rundle reported that TAO typically runs a biphasic course, which begins with rapid progressive inflammation that peaks after 6–24 months, before a chronic and fibrotic phase of varying duration [6]. Early treatment during the inflammatory phase is recommended to control the disease activity.

Glucocorticoid plays an important role to reduce inflammation in TAO, and it is was widely used as the first line of treatment of active TAO, and randomized controlled trials confirmed that the intravenous route was more effective than the oral route in reducing CAS, despite both being ineffective in reducing proptosis [12,22,23]. High-dose intravenous corticosteroid is the mainstay of treatment in moderate-to-severe and active TAO patients [13]. Inflammatory cell infiltrations occur in the active phase of the disease, which correlates with the disease activity. The 12-weekly IVMP regimen was reportedly effective in more than 70% of patients and was well tolerated [13]. In an earlier study, Bartalena et al. reported that the 7.47 gram IVMP regimen gave a transient advantage over the lower dose regimen, but with greater toxicity [14]. Later, He et al. reported that a 3-monthly of 1.5 gm IVMP was more effective in reducing ocular symptoms and disease activity with fewer recurrences, when compared to the 12-weekly (4.5 gm) regimen [16]. Zhu et al. found that the 12-weekly (4.5 gm) protocol of IVMP was more efficient and safer than the daily (total of 4.5 gm) protocol [25]. Recently, Mu et al. reported that both weekly and daily IVMP regimens of the same cumulative dosage (4.5 gm) showed comparable response rates [15].

High-dose intravenous glucocorticoid is the mainstay of treatment in moderate-to-severe and active TAO patients [13]. Inflammatory cells infiltration occurs during the active phase of TAO, which usually lasts for 6 months to 2 years, and the degree of infiltration correlates with the disease activity [8,9]. According to the EUGOGO clinical guidelines, 2 IVMP regimens were recommended together with mycophenolate sodium as the first-line treatment for moderate-to-severe, active TAO. However, until now, there is no activity or severity-driven individualized IVMP regimen in treating TAO. We suggest further clinical trials to define an activity-adjusted regimen for active TAO patients. Recent advances in the treatment of TAO include biological agents. Tocilizumab is a recombinant humanized IgG1 anti-interleukin 6 monoclonal antibody, and studies have shown that interleukin 6 increased thyroid-stimulating hormone receptor expression in fibroblasts. Tocilizumab is considered a viable treatment for TAO patients for whom IVMP is intolerable or ineffective [26,27].

CAS is a widely used assessment scale in evaluating the activity of TAO [19]. However, both the exophthalmometry values and upper eyelid signs are less prominent in the Asian population. Thus, CAS may be less sensitive among Asian patients when compared to Caucasian patients [28]. Despite that, our data still showed that patients with higher presenting CAS are more likely to receive additional treatment. Magnetic resonance imaging (MRI) has been reported as a reliable tool to differentiate active from inactive TAO. The Short Tau Inversion Recovery (STIR) sequences on MRI suppress the fat signals and highlight the fluid-filled tissues, which correlates well with the inflammatory orbital tissues [29,30]. Accurate evaluation of TAO activity is vital in clinical practice. We suggest further studies to understand the role of MRI in the activity-driven protocol for TAO patients.

High-dose IVMP has been used in systemic diseases such as multiple sclerosis, myasthenia gravis and spinal cord injury [31,32,33]. Common adverse effects include a change in sense of taste, facial flushing, stomachache/peptic ulcer, sleep disturbance and appetite change [34]. Marino et al. reported that acute liver damage occurred in 0.3% of TED patients following IVMP, and despite being statistically insignificant, the dose in patients who died was higher than in those who recovered [35]. We avoided consecutive daily high doses (≥1 g) and monitored the vital signs and liver function before and at regular intervals during IVMP. Our study did not show any difference in adverse event numbers between the 6-weekly and 12-weekly regimens. Both regimens were well-tolerated in our TAO patients, without severe adverse events.

The pathophysiology of TAO is complex and incompletely understood. A recent study reported a subset of orbital fibrocytes found in the orbit expressed high levels of the thyroid-stimulating hormone receptor (TSHR), and these cells can differentiate into myofibroblasts when stimulated, which are responsible for the soft tissue enlargement in TAO [36]. Insulin-like growth factor 1 (IGF-1) has also been implicated in the pathogenesis of TAO. IGF-1 receptors were also found to be highly expressed in orbital fibroblasts in TAO patients, and they are responsible for the activation of the fibroblasts and the production of extracellular matrix components such as glycosaminoglycans and hyaluronic acid [37,38,39]. The discovery of both TSHR and IGF-1 receptors has been found in orbital fibroblasts. The cross-talk between the two receptors showed synergistically increased hyaluronic acid secretion in the orbit. In addition, the synergistic increases in both potency and efficacy of TSH in the presence of IgF-1 were shown [40,41,42]. However, the exact autoimmune mechanism in the pathogenesis of TAO is still being elucidated.

There are several limitations to this study. Firstly, this is a retrospective comparative study with a sequential sampling of patients, with inherent differences in the documentation. Secondly, the sample size is relatively small; however, our data may provide a useful foundation of information for future prospective studies. Thirdly, this is a study in a single center for ethnic Han Chinese patients living in urbanized Hong Kong; future multi-center studies with patients of different ethnicities is suggested. Fourthly, CAS may be less sensitive in Asian patients, and further study using MRI to evaluate the disease activity in the Asian population is warranted.

In summary, we showed that those who did not require additional treatment after using a low dose 6-weekly IVMP regimen were associated with a lower presenting CAS. The low dose 6-weekly IVMP regimen is especially relevant in active patients with lower presenting CAS of less than 4. For patients with presenting CAS of 4 or above, a second 6-weekly course or the high dose 12-weekly IVMP regimen should be considered. Clinical trials are required to evaluate the use of activity-based individualized IVMP regimens for TAO patients.

## 6. Conclusions

Although less effective in improving CAS or exophthalmos than the high-dose 12-weekly IVMP regimen, the 6-weekly IVMP regimen is an acceptable treatment option in patients with marginal/lower CAS. Clinical trials are needed to define an activity or severity-based immunosuppression regimen in TAO.

## Figures and Tables

**Table 1:** The baseline information of TAO patients who received IVMP.

	6 Weekly Regimens	12 Weekly Regimens	*p* Values
No.	22 (44 eyes)	43 (86 eyes)	N/A
Age	54 ± 10	49 ± 12	0.0563
Sex (M:F)	7:15	17:26	0.464
Ethnicity	22(100%)	43(100%)	1.000
Smoker/ex-smoker	7(30%)	14(33%)	0.860
Time to Rx (Month)	7 ± 4	6 ± 3	0.412
History of Thyroid disease			
GD	21 (91%)	40(93%)	0.5422
RAI	2(9%)	6(14%)	0.335
Thyroidectomy	1(5%)	4(9%)	0.687
EGO	2 (9%)	3 (7%)	0.409
DON at presentation	1(4%)	0	0.339
Baseline clinical presentation			
CAS	3.6 ± 1.0	3.1 ± 0.9	0.304
BCVA	1.0 ± 0.4	1.0 ± 0.3	0.624
IOP(mmHg)	17.3 ± 5	17.5 ±5	0.469
MRD1(mm)	5.7 ± 2	4.8 ± 2	0.162
MRD2(mm)	5.6 ± 1	5.5 ± 1	0.335
Exophthalmos (mm)	19.1 ± 3	19.7 ± 4	0.313
EOMy	1.5 ± 2	1.18 ± 2	0.595
Diplopia	0.4 ± 0.8	0.8 ± 0.6	0.190

Legends: Age = Age of TAO onset, EGO = euthyroid Graves’ ophthalmopathy, F = female, GD = Graves’ disease, IVMP = Intravenous pulse methylprednisolone, M = Male, mm = millimeter, mmHg = millimeter of mercury, N/A = Not applicable, No = Number of patients, NS = Not significant, RAI = Radioactive iodine treatment, TAO = Thyroid-associated orbitopathy.

**Table 2:** The comparisons of treatment outcomes between the 6-weekly and 12-weekly IVMP regimens.

	6-Weekly	12-Weekly	*p* Value
No. (patients)	22	43	
CAS			
Baseline	3.6	3.1	N/A
0–6th (weeks)	−0.8	−1.5	0.0279 *
0–12th	−1.1	−2.1	0.00228 *
0–26th	−2.2	−2.1	0.312
0–52th	−1.2	−2.2	0.0228 *
Diplopia			
Baseline	0.4	0.8	N/A
0–6th (weeks)	+0.03	+0.03	0.338
0–12th	+0.1	+0.03	0.145
0–26th	−0.04	+0.2	0.376
0–52th	+0.2	+0.1	0.384
Needs of additional IVMP+/−ORT	15(65%)	11 (26%)	0.00169 *
No. (eyes)	44	86	
BCVA			
Baseline	1.0	1.0	N/A
0–6th (weeks)	+0.1	−0.07	0.350
0–12th	+0.09	−0.01	0.0736
0–26th	+0.05	−0.06	0.303
0–52th	+0.1	+0.004	0.050
IOP (mmHg)			
Baseline	16.1	17.3	N/A
0–6th (weeks)	+0.1	−0.6	0.284
0–12th	+0.1	−0.04	0.111
0–26th	+0.3	+1.1	0.0570
0–52th	+0.5	−0.6	0.121
MRD1(mm)			
Baseline	5.7	4.8	N/A
0–6th (weeks)	−0.4	+0.2	0.425
0–12th	−0.1	−0.1	0.494
0–26th	−0.2	+0.2	0.365
0–52th	−1.2	−0.4	0.490
MRD2(mm)			
Baseline	5.3	5.4	N/A
0–6th (weeks)	−0.2	−0.1	0.435
0–12th	−0.09	−0.1	0.486
0–26th	+0.2	+0.5	0.294
0–52th	+0.1	+0.05	0.369
Exophthalmos (mm)			
Baseline	18.6	19.3	N/A
0–6th (weeks)	+0.1	−0.7	0.0453 *
0–12th	+0.5	−0.8	0.0347 *
0–26th	−0.5	−0.05	0.277
0–52th	−0.8	−0.7	0.259
EOMy			
Baseline	1.5	1.2	N/A
0–6th (weeks)	−0.2	0	0.453
0–12th	−0.2	−0.2	0.234
0–26th	−0.1	0	0.234
0–52th	−0.1	−0.1	0.232

Legends: Age = Age of TAO onset, EGO = Euthyroid Graves’ ophthalmopathy, F = female, GD = Graves’ disease, IVMP = Intravenous pulse methylprednisolone, M = Male, mm = millimeter, mmHg = millimeter of mercury, N/A = Not applicable, No = Number of, NS = Not significant, RAI = Radioactive iodine treatment, TAO = Thyroid associated orbitopathy, * = *p* < 0.05.

**Table 3:** Comparisons of the parameters among patients who required and not required additional treatment in the 6-weekly IVMP group.

	Without Additional Rx	With Additional Rx	*p* Value
N	8	14	N/A
Age	55+/−10	53+/−12	0.253
Sex (F:M)	6:2	9:5	0.678
CAS	3 ± 0	4 ± 1	0.0193 *
BCVA	1.0 ± 0.4	0.9 ± 0.3	0.407
IOP(mmHg)	19 ±5	16 ± 5	0.106
MRD1(mm)	6 ± 2	5 ± 2	0.172
Exophthalmos (mm)	18 ± 4	19 ± 4	0.268
EOMy	−1.5 ± 3	1.7 ± 2	0.441
Diplopia	0.5 ± 0.5	0.4 ± 0.5	0.410

Legends: Age = Age of TAO onset, F = female, GD = Graves’ disease, IVMP = Intravenous pulse methylprednisolone, M = Male, mm = millimeter, mmHg = millimeter of mercury, N/A = Not applicable, No = Number of patients, NS = Not significant, RAI = Radioactive iodine treatment, TAO = Thyroid associated orbitopathy, * = *p* < 0.05.

**Table 4:** The comparisons of adverse events from the 6-weekly and 12-weekly IVMP regimens.

	6-Weekly	12-Weekly
N	22	43
Transient epigastric pain	2	1
Nausea and vomiting	2	1
Facial flushing	2	0
Palpitation	1	0
Insomnia	0	3
Elevated ALP	0	0
Deranged lipid profile	0	0
Elevated fasting glucose	0	0

Legends: IVMP = Intravenous pulse methylprednisolone, N/A = Not applicable, No = Number of patients.

## Data Availability

All data generated or analyzed during this study are included in this article. Further enquiries can be directed to the corresponding author.

## Abbreviations

CAS = Clinical activity score, DON = Dysthyroid optic neuropathy, IVMP = Intravenous methylprednisolone, ORT = Orbital radiotherapy, SD = Standard deviation, TAO = Thyroid-associated orbitopathy.
